# Pretreatment comprehensive nutritional index predicts survival in locally advanced rectal cancer after neoadjuvant chemoradiotherapy and surgery

**DOI:** 10.17305/bb.2026.13609

**Published:** 2026-01-30

**Authors:** Zhexue Wang, Liming Zhao, Pu Cheng, Mandula Bao, Fei Huang, Ruoxi Tian, Jiyun Li, Hengchang Liu, Zhaoxu Zheng

**Affiliations:** 1 Department of Colorectal Surgery, National Cancer Center/National Clinical Research Center for Cancer/Cancer Hospital, Chinese Academy of Medical Sciences and Peking Union Medical College, Beijing, China

**Keywords:** Comprehensive nutritional index, nutritional status, neoadjuvant chemoradiotherapy, prognosis, locally advanced rectal cancer

## Abstract

Nutritional status significantly influences treatment tolerance and long-term outcomes in patients with locally advanced rectal cancer (LARC); however, individual nutritional markers may not fully capture overall nutritional reserves. This study aimed to evaluate the prognostic value of a comprehensive nutritional index (CNI), derived from principal component analysis, in patients with LARC undergoing neoadjuvant chemoradiotherapy (NCRT) followed by surgical intervention. We conducted a retrospective analysis of 336 patients with LARC who received NCRT followed by surgery between 2014 and 2019. The CNI was constructed using body mass index, usual body weight percentage, total lymphocyte count, serum albumin, and hemoglobin levels. Patients were categorized into low- and high-CNI groups based on an outcome-oriented cut point, and survival outcomes were assessed through Kaplan–Meier analysis and Cox regression. Patients with lower CNI scores exhibited significantly poorer overall survival and disease-free survival compared to those with higher CNI scores. Furthermore, CNI remained independently associated with both endpoints after adjusting for established pathological factors, including tumor regression grade and ypN stage. A nomogram that integrates CNI, tumor regression grade, and ypN stage demonstrated favorable discrimination and calibration during internal validation. These findings support the use of pretreatment CNI as a practical nutritional composite associated with prognosis in LARC patients treated with NCRT, and the proposed nomogram may enhance individualized risk estimation.

## Introduction

Colorectal cancer (CRC) is one of the most prevalent malignancies globally and continues to present a significant disease burden despite advancements in screening and treatment [1]. Locally advanced rectal cancer (LARC) represents a major subset of CRC, characterized by heightened risks of recurrence and metastasis, thereby necessitating the optimization of prognostic evaluation and treatment strategies as a critical clinical challenge [2].

In recent years, the treatment paradigm for LARC has evolved from surgery alone to a multidisciplinary integrated approach. Neoadjuvant chemoradiotherapy (NCRT) followed by total mesorectal resection has emerged as the standard therapeutic strategy for LARC patients, enhancing resectability and local control [3, 4]. However, tumor responses to NCRT are notably heterogeneous, and LARC patients continue to experience severe complications and poor prognoses [5, 6]. Consequently, the utilization of preoperative clinical parameters for more accurate prognosis prediction is essential.

Malnutrition is prevalent among rectal cancer patients due to tumor burden, treatment toxicity, and metabolic alterations, and is closely associated with increased complications, diminished treatment tolerance, and poorer survival outcomes [7]. Conventional nutritional markers, such as body mass index (BMI), serum albumin (ALB), and the prognostic nutritional index (PNI), provide only a partial reflection of a patient’s systemic condition [8–10]. The Comprehensive Nutritional Index (CNI), derived from five nutrition-related indicators—BMI, usual body weight percentage (UBWP), total lymphocyte count (TLC), ALB, and hemoglobin (HB)—integrates anthropometric and laboratory parameters into a composite score. Recent studies have underscored the prognostic significance of CNI across various malignancies, including LARC patients undergoing NCRT, indicating that CNI may serve as a reliable indicator of systemic nutritional and immune status [11–15].

Nevertheless, further independent validation of CNI within larger, well-characterized cohorts, as well as exploration of its integration with established pathological prognostic factors, is warranted. Therefore, this study aims to independently validate the prognostic value of CNI in LARC patients treated at a high-volume cancer center and to develop a CNI-based prognostic model by integrating pathological response and nodal status, with the objective of enhancing postoperative risk stratification and clinical applicability.

## Materials and methods

### Patients

This retrospective analysis included 336 LARC patients treated at our institution between September 2014 and July 2019. All patients had histologically confirmed rectal adenocarcinoma and underwent standardized long-course NCRT followed by total mesorectal excision. Initially, 978 patients with LARC were screened during the study period. Exclusion criteria included lack of neoadjuvant therapy, non-adenocarcinoma histology, treatment with neoadjuvant chemotherapy or radiotherapy alone, history of other malignancies, implementation of a watch-and-wait strategy post-neoadjuvant treatment, surgery performed at external institutions, incomplete key clinicopathological data, or loss to follow-up. Ultimately, 336 patients who completed standard NCRT followed by radical surgery and had complete data were included in the final analytical cohort. This study was a retrospective cohort analysis based on routinely collected clinical data. All patient information was anonymized before analysis, with no additional interventions or patient contact involved. In accordance with institutional policy and national regulations, this type of retrospective analysis using de-identified data was exempt from formal ethics committee approval, and the requirement for informed consent was waived.

### Patient selection

From 2014–2019, 978 patients with rectal cancer were screened. After excluding those without NCRT (*n* ═ 596), non-adenocarcinoma histology (*n* ═ 3), non-standard neoadjuvant treatment (*n* ═ 23), a history of other malignancies (*n* ═ 9), and missing key clinical data or follow-up (*n* ═ 11), 336 patients were ultimately included in the study.

### Clinicopathological data

Clinicopathological data were collected, encompassing age, gender, smoking and drinking history, hypertension, diabetes, BMI, serum carcinoembryonic antigen (CEA) and cancer antigen (CA) 19–9 levels, T-stages and N-stages evaluated via magnetic resonance imaging (MRI), vessel invasion, perineural invasion, and post-neoadjuvant pathological T stage (ypT) and post-neoadjuvant pathological N stage (ypN) stages assessed by histopathology. Tumor regression grade (TRG) was evaluated on postoperative surgical specimens using the Dworak grading system [16], which classifies the tumor response to NCRT into five levels based on the proportion of residual tumor cells and the extent of fibrosis. In this system, TRG 0 indicates no evidence of regression, TRG 1 reflects minimal tumor response, TRG 2 denotes moderate regression with a mixture of fibrosis and residual tumor, TRG 3 represents marked regression with only small clusters of viable cells, and TRG 4 corresponds to complete tumor regression with no residual carcinoma. For analytical purposes, patients were categorized into two groups based on their pathological response: those with TRG 3–4 were classified as having a good response, while those with TRG 0–2 were considered to have a poor response. This dichotomization facilitated a clearer comparison of treatment outcomes within the study cohort.

## NCRT

All patients received long-course NCRT according to a standardized institutional protocol. Pelvic radiotherapy was administered with a total dose of 45–50.4 Gy in 25–28 fractions. Concurrent chemotherapy consisted of fluoropyrimidine-based regimens, including continuous-infusion 5-fluorouracil or oral capecitabine, during radiotherapy.

### Follow-up

Postoperative surveillance for all patients was conducted at regular intervals, with follow-up visits scheduled every three months during the first postoperative year and every six months thereafter for a minimum of three years. The primary survival endpoints were overall survival (OS) and disease-free survival (DFS). OS was defined as the interval from the date of surgery to death attributable to rectal cancer or to the most recent follow-up for patients who were still alive. DFS was defined as the time from surgical resection to tumor recurrence. Follow-up assessments were conducted through outpatient clinic visits or telephone interviews.

### Calculation of nutritional status

CNI was calculated based on previous research, utilizing five individual nutritional indicators: HB, TLC, BMI, ALB, and UBWP, through principal component analysis (PCA). In the PCA framework, the coefficients (loadings) represent the contribution of each standardized variable to the derived components rather than independent prognostic effects. Thus, the direction of an individual loading (positive or negative) should be interpreted contextually within the broader multivariate nutritional pattern captured by the CNI, rather than as a direct inverse clinical association. All nutritional parameters, including BMI, UBWP, TLC, serum ALB, and HB, were uniformly assessed within one week prior to the initiation of NCRT. BMI was calculated as weight (kg)/height (m)^2^, while UBWP was defined as the ratio of current body weight (CBW) to a reference body weight, expressed as a percentage. In this study, CBW referred to the body weight recorded prior to the start of NCRT. The reference body weight, previously referred to as usual body weight (UBW), was estimated using the height-based Lorentz formula: for women, [height (cm) -- 100] -- [height (cm) -- 150]/2.5; and for men, [height (cm) -- 100] -- [height (cm) -- 150]/4. This calculated value represents a theoretical reference weight rather than a historically measured body weight. The nutrition risk index (NRI) was calculated using the equation: 1.519 × ALB (g/L) + 41.7 × (CBW / ideal body weight). Ideal body weight (IBW) was estimated as 22 × height^2^ (m^2^). The PNI was calculated using the formula: ALB (g/L) + 0.005 × TLC (µL). Correlations among nutritional indicators were assessed using Pearson correlation analysis and visualized with a heatmap.

### Statistical analysis

The CNI was constructed using PCA based on five nutritional indicators. Components were retained to ensure that cumulative explained variance exceeded 80%. The suitability of the data for PCA was assessed using the Kaiser-Meyer-Olkin (KMO) measure and Bartlett’s test of sphericity, with a KMO value greater than 0.60 and a significant Bartlett’s test deemed acceptable. The optimal CNI cut-off was determined through an outcome-oriented approach focusing on overall survival, aimed at maximizing the separation of survival curves rather than relying on a distribution-based threshold. Based on this cut-off, patients were categorized into low- and high-CNI groups, with this categorical CNI variable utilized in subsequent Cox regression analyses and nomogram development. Categorical variables were compared using the chi-square test or Fisher’s exact test as appropriate. Continuous variables were assessed for normality using the Shapiro-Wilk test; normally distributed variables were analyzed with Student’s *t*-test, while non-normally distributed variables were examined using non-parametric methods. Cox proportional hazards regression analyses were conducted to identify prognostic factors for OS and DFS. The proportional hazards assumption was evaluated using Schoenfeld residuals, revealing no significant violations. CNI was initially analyzed as a continuous variable for exploratory purposes, and restricted cubic spline analyses were employed to explore potential non-linear associations with survival outcomes. In addition to CNI, other nutritional indices, including the PNI and NRI, were calculated and evaluated in univariate Cox analyses for contextual comparison; CNI was predefined as the primary nutritional index, while analyses of other indices were considered exploratory. Multivariable Cox models incorporated a limited number of covariates selected a priori based on clinical relevance and univariate significance. A prognostic nomogram was developed using variables independently associated with outcomes. Internal validation was conducted utilizing 400 bootstrap resamples with replacement. Model discrimination was primarily assessed using the concordance index (C-index) and time-dependent receiver operating characteristic (ROC) analyses at prespecified time points. Calibration curves and decision curve analysis (DCA) were employed to evaluate model calibration and clinical utility. All analyses were executed using R software (version 4.3.4), with a two-sided *P* value less than 0.05 deemed statistically significant.

Following the determination of the optimal cut-off value, CNI was treated as a categorical variable (low vs. high) and utilized in subsequent Cox regression analyses and nomogram development.

## Results

### Patient characteristics

A total of 336 patients with LARC were included in the study. The mean age of participants was 56.40 ± 10.82 years, with 66.7% being male. The average BMI was 24.07 ± 3.22 kg/m^2^. A history of smoking and alcohol consumption was reported in 39.0% and 34.5% of patients, respectively. Hypertension was present in 28.6% of the cohort, while 14.9% had diabetes. In terms of clinical staging, 15.5% of patients were classified as clinical tumor–node–metastasis stage (cTNM) stage II and 84.5% as stage III. Postoperative pathological staging revealed that 18.5% had post-neoadjuvant pathological tumor–node–metastasis stage (ypTNM) stage I, 48.5% stage II, and 33.0% stage III. Complete tumor regression (TRG 4, pathological complete response) was observed in 53 patients, categorized into the good-response group (TRG 3–4). The median follow-up duration was 49 months (range, 8–90 months). The baseline characteristics of the cohort are summarized in Table 1.

**Table 1:** Baseline characteristics of patients

**Characteristics**	***n* (%)/mean ± *SD***
Age (years)	56.40±10.82
Gender (female/male)	112 (33.3)/224 (66.7)
BMI	24.07±3.22
Smoking history (yes/no)	131 (39.0)/205 (61.0)
Drinking history (yes/no)	116 (34.5)/220 (65.5)
Hypertension (yes/no)	96 (28.6)/240 (71.4)
Diabetes (yes/no)	50 (14.9)/286 (85.1)
cTNM (II/III)	52 (15.5)/283 (84.5)
ypTNM (I/II/III)	62 (18.5)/163 (48.5)/111 (33.0)
Vessel invasion (yes/no)	32 (9.5)/304 (90.5)
Perineural invasion (yes/no)	52 (15.5)/284 (84.5)

Note: Due to the absence of clinical T stage data for one patient, the total count for the cTNM stage does not equal 336. Abbreviations: BMI: Body mass index; cTNM: Clinical tumor–node–metastasis stage; ypTNM: Postoperative pathological tumor–node–metastasis stage.

### Construction of CNI

The KMO measure indicated adequate sampling adequacy, and Bartlett’s test of sphericity was statistically significant (*P* < 0.001), justifying the use of PCA. PCA was applied to five nutritional indicators: TLC, BMI, ALB, HB, and UBWP. The first three principal components (PCs) were retained based on the criterion that cumulative explained variance exceeded 80%. These PCs were derived as linear combinations of the standardized nutritional variables. Specifically, PC1 was defined as 0.693 × Y1 + 0.663 × Y2 + 0.039 × Y3 + 0.098 × Y4 + 0.165 × Y5; PC2 as --0.134 × Y1 -- 0.139 × Y2 + 0.356 × Y3 + 0.615 × Y4 + 0.678 × Y5; and PC3 as --0.011 × Y1 -- 0.015 × Y2 -- 0.875 × Y3 + 0.484 × Y4 + 0.016 × Y5. The CNI was subsequently computed by combining these three components according to their respective variance contributions, using the formula: CNI = 0.406 × PC1 + 0.265 × PC2 + 0.198 × PC3. By substituting the component loadings into this expression, the CNI can also be represented as a weighted sum of the original standardized variables: CNI = 0.244 × Y1 + 0.229 × Y2 -- 0.063 × Y3 + 0.299 × Y4 + 0.250 × Y5, where Y1-Y5 represent normalized TLC, BMI, ALB, HB, and UBWP, respectively. Specifically, BMI was measured in kg/m^2^, UBWP as a percentage (%), TLC as cells per microliter (cells/µL), serum albumin as g/L, and hemoglobin as g/L prior to normalization.

### Association between continuous CNI and survival outcomes

**Figure S1.**
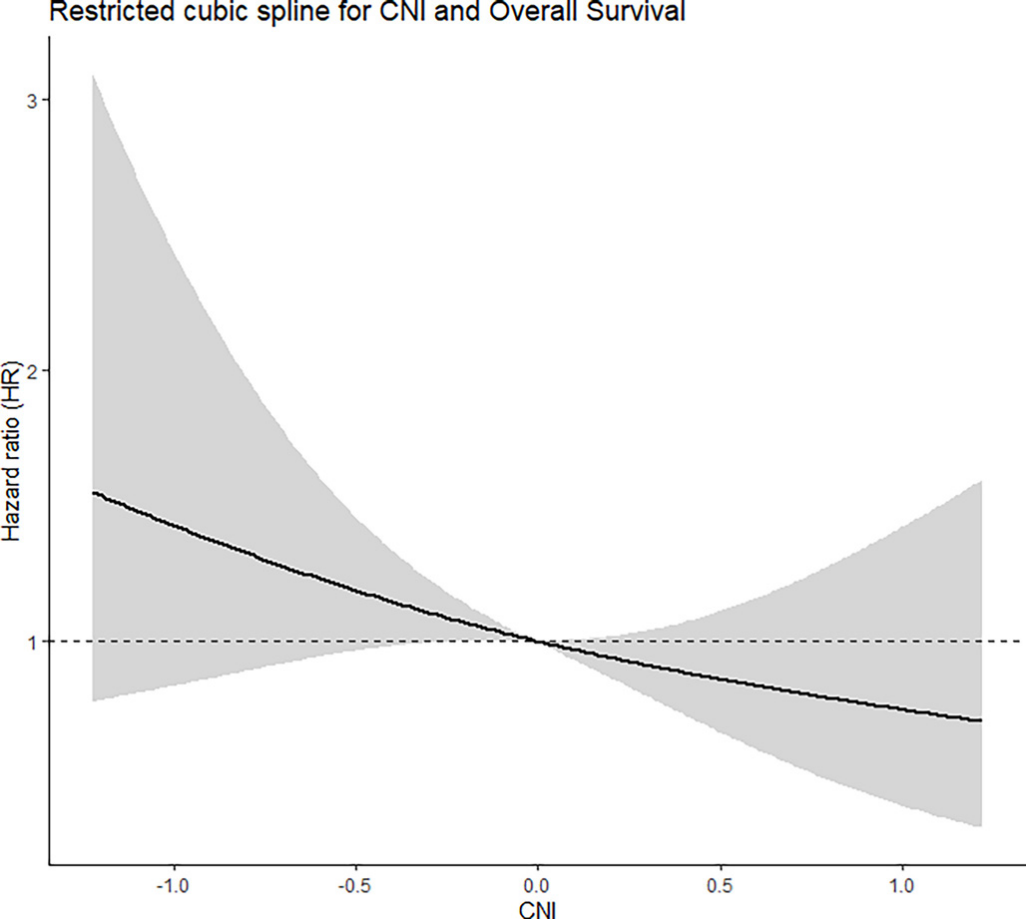
**Association between continuous CNI and OS using restricted cubic spline analysis.** Restricted cubic spline plot from an adjusted Cox proportional hazards model illustrating the relationship between continuous CNI and OS. The solid curve shows the adjusted HR across CNI values, and the shaded band denotes the 95% CI. The dashed horizontal line indicates the reference HR of 1.0, anchored at the median CNI value. The curve demonstrates an approximately linear inverse association between CNI and the risk of death, with no clear evidence of non-linearity. Abbreviations: CNI: Comprehensive nutritional index; OS: Overall survival; HR: Hazard ratio; CI: Confidence interval.

When modeled as a continuous variable, higher CNI values were significantly associated with improved OS in Cox regression analysis. Restricted cubic spline analysis indicated an approximately linear inverse relationship between CNI and the risk of death, with no clear evidence of a non-linear association. These findings support the robustness of CNI as a continuous prognostic indicator (Figure S1).

### Prognostic stratification using the CNI

**Figure 1.**
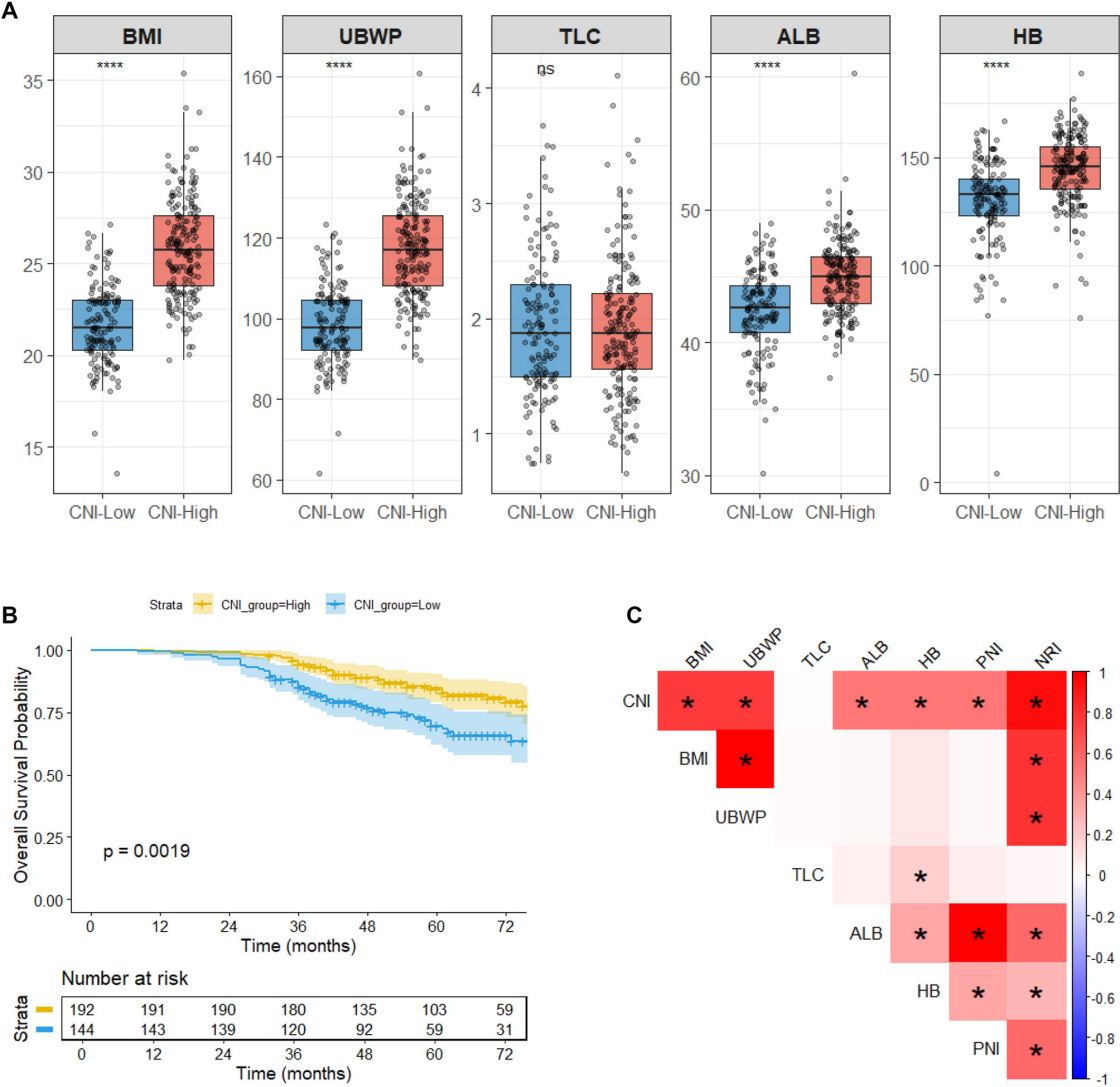
**Nutritional characteristics, OS, and correlations stratified by CNI.** (A) Pretreatment levels of BMI, UBWP, TLC, ALB, and HB by CNI group (box-and-whisker plots with individual datapoints). (B) Kaplan–Meier OS curves by CNI group; shaded bands indicate 95% CIs and numbers at risk are shown below (log-rank *P* ═ 0.0019). (C) Pearson correlation heatmap showing associations between CNI and nutritional indicators/indices (BMI, UBWP, TLC, ALB, HB, PNI, and NRI); the color scale represents Pearson correlation coefficients (r), and asterisks denote statistically significant correlations (*P* < 0.05). CNI groups were defined using an outcome-oriented cut point of 0.3879: Low CNI (CNI < 0.3879, *n* ═ 144) and high CNI (CNI ≥ 0.3879, *n* ═ 192). In panel A, significance labels indicate between-group differences (****P < 0.0001; ns, not significant). Abbreviations: CNI: Comprehensive nutritional index; BMI: Body mass index; UBWP: Usual body weight percentage; TLC: Total lymphocyte count; ALB: Albumin; HB: Hemoglobin; OS: Overall survival; CI: Confidence interval; PNI: Prognostic nutritional index; NRI: Nutritional risk index.

The median pretreatment CNI for the entire cohort was --0.0087. Using an outcome-oriented optimal cut-off value of 0.3879 identified by the surv_cutpoint function in R software, patients were stratified into a low-CNI group (CNI < 0.3879, *n* ═ 144) and a high-CNI group (CNI ≥ 0.3879, *n* ═ 192). The ROC for OS yielded an area under the curve (AUC) of 0.81, demonstrating a statistically significant association between CNI and survival outcomes (*P* < 0.05). As illustrated in Figure 1A, baseline nutritional characteristics differed significantly between the two groups. Baseline clinicopathological characteristics stratified by CNI group are summarized in Table S1. Importantly, key tumor-related baseline factors, including clinical TNM stage, pathological TNM stage, vessel invasion, and perineural invasion, were well balanced between the low- and high-CNI groups. Patients in the high-CNI group exhibited markedly superior levels of key nutritional parameters, including BMI, UBWP, ALB, and HB. Kaplan-Meier survival analysis demonstrated significantly poorer OS in the low-CNI group compared to the high-CNI group (*P* ═ 0.0019; Figure 1B). Accordingly, CNI was evaluated both as a continuous variable and as a categorical variable in Cox regression analyses, with the categorized CNI used for multivariable modeling and nomogram construction to enhance clinical interpretability. Among the nutritional indices evaluated, CNI, predefined as the primary index, demonstrated a significant association with OS. This superior predictive performance was further confirmed by univariate Cox regression analysis presented in Table 2. Among all nutritional indices evaluated, CNI exhibited the most potent protective effect on OS (HR = 0.697, 95% CI: 0.497–0.978, *P* ═ 0.037). The correlations between CNI and its constituent nutritional indicators are visualized in Figure 1C.

**Table 2:** Univariate Cox regression results for nutritional indices

**Variable**	**HR (95% CI)**	***P* value**
BMI	0.906 (0.843–0.973)	0.007
UBWP	0.978 (0.963–0.994)	0.007
TLC	0.782 (0.535–1.141)	0.202
ALB	0.956 (0.891–1.027)	0.218
HB	0.992 (0.981–1.003)	0.165
CNI	0.697 (0.497–0.978)	0.037
NRI	0.960 (0.933–0.988)	0.005
PNI	0.955 (0.907–1.006)	0.082

Abbreviations: HR: Hazard ratio; CI: Confidence interval; BMI: Body mass index; UBWP: Usual body weight percentage; TLC: Total lymphocyte count; ALB: Albumin; HB: Hemoglobin; CNI: Comprehensive nutritional index; NRI: Nutritional risk index; PNI: Prognostic nutritional index.

**Figure 2.**
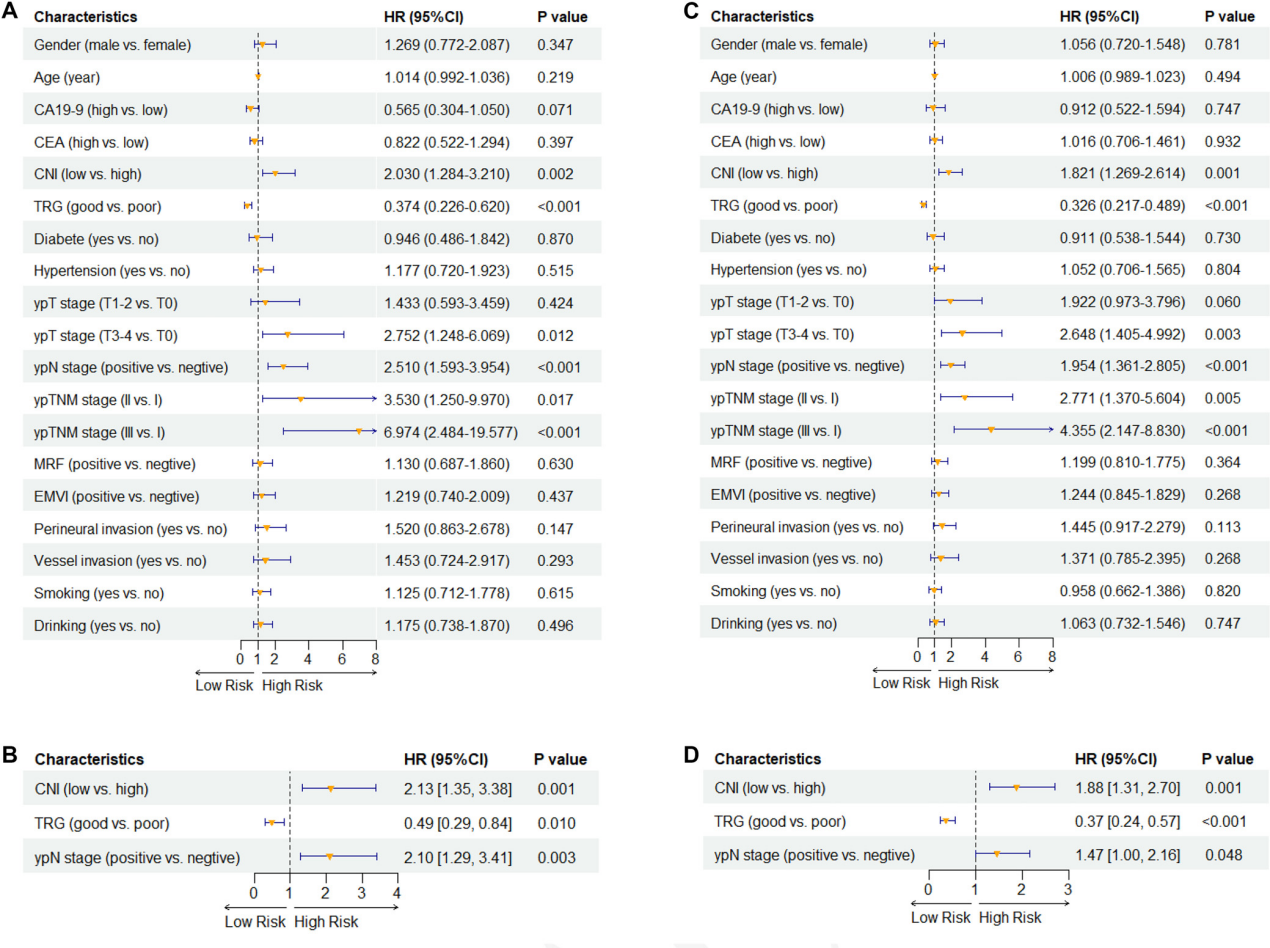
**Cox regression analyses for OS and DFS.** (A) Univariate Cox proportional hazards analysis for OS. (B) Multivariate Cox analysis for OS including CNI, TRG, and ypN stage. (C) Univariate Cox proportional hazards analysis for DFS. (D) Multivariate Cox analysis for DFS including CNI, TRG, and ypN stage. CNI was analyzed as a categorical variable using the predefined cut point (low vs high; CNI < 0.3879 vs CNI ≥ 0.3879). In univariate analyses, low CNI was associated with worse OS (HR = 2.03, 95% CI: 1.28–3.21; *P* ═ 0.002) and DFS (HR = 2.10, 95% CI: 1.35–3.26; *P* ═ 0.001). After adjustment for TRG and ypN stage, low CNI remained independently associated with worse OS (HR = 2.13, 95% CI: 1.36–3.35; *P* ═ 0.001) and DFS (HR = 1.88, 95% CI: 1.20–2.94; *P* ═ 0.001). Points indicate HRs and horizontal bars indicate 95% CIs; the dashed vertical line denotes HR = 1. Abbreviations: OS: Overall survival; DFS: Disease-free survival; HR: Hazard ratio; CI: Confidence interval; CNI: Comprehensive nutritional index; TRG: Tumor regression grade; ypN: Post-therapy pathologic regional lymph node stage; CEA: Carcinoembryonic antigen; CA19-9: Carbohydrate antigen 19-9; MRF: Mesorectal fascia; EMVI: Extramural vascular invasion; ypT: Post-therapy pathologic primary tumor stage; ypTNM: Postoperative pathological tumor–node–metastasis stage.

**Figure 3.**
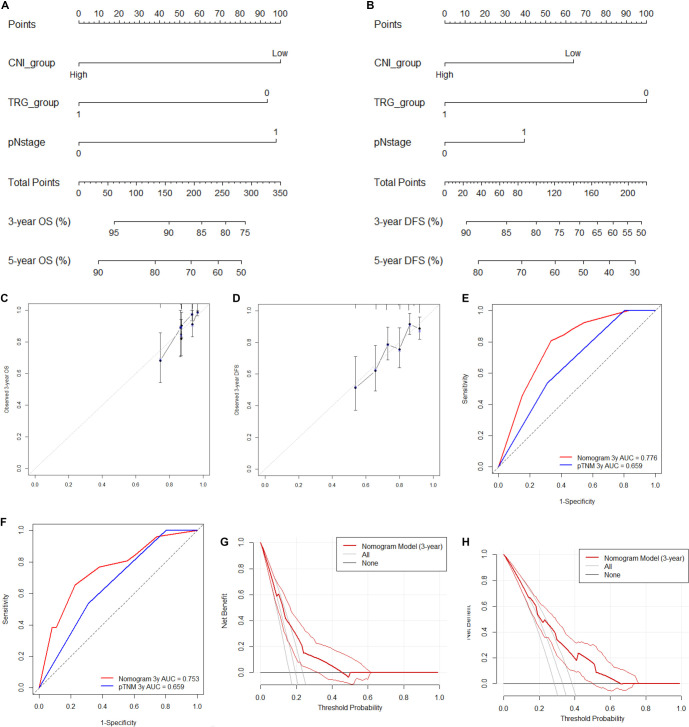
**Development and internal validation of CNI-based nomograms for OS and DFS.** (A) Nomogram incorporating categorized CNI, TRG, and ypN stage to estimate 3-year and 5-year OS. (B) Nomogram incorporating categorized CNI, TRG, and ypN stage to estimate 3-year and 5-year DFS. (C) Calibration plots for 3-year and 5-year OS showing agreement between predicted probabilities and observed outcomes following internal validation with bootstrap resampling (*n* ═ 400); the diagonal line represents ideal calibration. (D) Calibration plots for 3-year and 5-year DFS following bootstrap internal validation (*n* ═ 400). (E) Time-dependent ROC curves at 3 years comparing discrimination of the nomogram versus ypTNM stage alone for OS. (F) Time-dependent ROC curves at 3 years comparing discrimination of the nomogram versus ypTNM stage alone for DFS. (G) DCA at 3 years for OS demonstrating net clinical benefit of the nomogram across a range of threshold probabilities compared with treat-all and treat-none strategies. (H) DCA at 3 years for DFS demonstrating net clinical benefit of the nomogram across a range of threshold probabilities. Abbreviations: CNI: Comprehensive nutritional index; TRG: Tumor regression grade; ypN: Post-therapy pathologic regional lymph node stage; OS: Overall survival; DFS: Disease-free survival; ROC: Receiver operating characteristic; DCA: Decision curve analysis; ypTNM: Postoperative pathological tumor–node–metastasis stage.

### CNI as an independent prognostic factor for OS and DFS

During the follow-up period, a total of 75 deaths and 120 events of disease recurrence or death were recorded. To assess the independent prognostic value of the CNI, both univariate and multivariate Cox regression analyses were conducted for OS and DFS. The CNI was categorized as low vs high based on a predefined cut-off value for inclusion in the survival analyses. In the univariate analysis (Figures 2A and C), a low CNI was significantly associated with poorer OS (HR = 2.03, 95% CI: 1.28–3.21, *P* ═ 0.002) and DFS (HR = 2.10, 95% CI: 1.35–3.26, *P* ═ 0.001). Other significant factors associated with outcomes included poor TRG and positive ypN stage. Subsequently, multivariate Cox analyses were performed, adjusting for these significant clinicopathological factors. The categorized CNI remained an independent prognostic factor for both OS (HR = 2.13, 95% CI: 1.36–3.35, *P* ═ 0.001) and DFS (HR = 1.88, 95% CI: 1.20–2.94, *P* ═ 0.001), alongside TRG and ypN stage (Figures 2B and D).

### Development and validation of a prognostic nomogram

To translate the independent prognostic factors (categorized CNI, TRG, and ypN stage) into a practical tool, nomograms were developed to predict OS and DFS, respectively (Figures 3A and B). Internal validation was conducted using bootstrap resampling (*n* ═ 400), demonstrating good agreement between predicted and observed outcomes as illustrated by the calibration curves (Figures 3C and D).

Nomogram discrimination was primarily assessed using the concordance index (C-index). The optimism-corrected C-index of the nomogram for OS was 0.677, indicating acceptable discriminative ability. Calibration plots showed good concordance between predicted and observed survival probabilities, with no significant deviation from the ideal line. Time-dependent ROC analyses provided a descriptive evaluation of nomogram performance (Figures 3E and F). Additionally, DCA indicated the potential clinical utility of the nomogram across various threshold probabilities (Figures 3G and H).

## Discussion

Nutritional status is pivotal in the treatment and prognosis of patients with rectal cancer, as inadequate nutrition can diminish treatment tolerance and elevate the risk of adverse events [17, 18]. Identifying patients at nutritional risk may thus facilitate early intervention and improve long-term outcomes. In this study, we evaluated the CNI, derived from BMI, UBWP, TLC, ALB and HB, to examine its prognostic value in patients with LARC treated with NCRT. The CNI exhibited superior prognostic performance compared to traditional nutritional indices in predicting survival outcomes. Although the CNI encompasses multiple nutritional and immune-related parameters, this study was not designed for formal head-to-head comparisons with established nutritional indices. Therefore, while the CNI demonstrated independent prognostic value, caution is advised when interpreting its relative performance compared to other nutritional scores. Patients with low CNI exhibited significantly worse OS and DFS than those with higher CNI values. Furthermore, the CNI remained an independent prognostic factor after adjusting for TRG and ypN stage. These findings suggest that the CNI captures multiple aspects of host status and provides more reliable prognostic information than individual nutritional indicators in this context. It is noteworthy that the cut-off value for CNI stratification was derived from the same cohort using an outcome-oriented approach. Based on this cut-off, the CNI was operationalized as a dichotomous variable and incorporated into multivariable Cox regression analyses and nomogram construction to enhance clinical interpretability. However, as this threshold was outcome-derived within the same cohort, the effect estimates from cut-off–based modeling may be subject to optimism.

In recent years, the CNI has garnered increasing attention as a prognostic marker across various malignant and chronic diseases [19]. Previous studies in nasopharyngeal carcinoma (NPC) demonstrated that lower CNI values correlate with more advanced disease stages and poorer survival outcomes [12, 13]. These investigations further suggested that the CNI may provide stronger prognostic information than conventional nutritional indices, such as the NRI and PNI, in this patient population. Additionally, low CNI has been associated with impaired overall nutritional status and diminished quality of life among NPC patients [13, 14]. Beyond NPC, evidence from hepatocellular carcinoma cohorts treated with transcatheter arterial chemoembolization indicates that patients with lower CNI values are more likely to experience severe treatment-related complications and poorer prognoses [20]. A recent study reported that a processed CNI derived from multiple nutrition-related parameters serves as a sensitive and reliable predictor of treatment response, postoperative morbidity, and survival outcomes in patients with esophageal squamous cell carcinoma undergoing neoadjuvant immunotherapy combined with chemotherapy [11]. In this study, the CNI demonstrated stronger prognostic capability than its individual components and outperformed traditional indices. This suggests that the CNI may provide a more reliable method for assessing nutritional status and treatment tolerance in patients receiving NCRT for LARC.

BMI, UBWP, ALB, TLC, and HB are routinely used clinical parameters that capture complementary dimensions of nutritional and physiological condition. BMI reflects overall body composition and nutritional reserve, with lower values commonly associated with malnutrition, sarcopenia, and metabolic imbalance [21, 22]. Previous studies have shown that a reduced preoperative BMI correlates with unfavorable outcomes in patients with gastrointestinal malignancies [23]. UBWP represents recent changes in body weight and may indicate protein-energy deficiency. ALB is widely regarded as an indicator of protein stores and systemic nutritional status; however, while hypoalbuminemia has been associated with postoperative complications, its prognostic significance and responsiveness to nutritional intervention remain debated [24–26]. TLC reflects immune competence, particularly cell-mediated immunity, which can be compromised by malnutrition. Reduced immune function adversely affects cancer prognosis, underscoring the relevance of TLC as a marker of host defense and tumor surveillance [27, 28]. HB reflects chronic protein status and has been associated with outcomes in several gastrointestinal cancers [29, 30]. Many clinicians rely on single nutritional parameters, but these isolated markers only capture part of the patient’s condition and often yield inconsistent results. Although TLC is generally regarded as a marker of immune and nutritional status, its loading in the PCA-derived CNI was negative in this study, and no significant difference in TLC was observed between the high- and low-CNI groups. This finding does not imply that a higher TLC is associated with adverse outcomes, but rather reflects the correlations among the included nutritional variables within a multivariate PCA framework after standardization. Within this composite model, TLC may therefore provide limited incremental discriminatory information beyond other nutritional indicators. Importantly, this observation suggests that while the current CNI is prognostically informative, there remains potential for further refinement. Future research may explore alternative combinations of nutritional and immune-related markers to optimize composite indices and enhance both clinical interpretability and predictive performance.

Composite indices such as the PNI and NRI aim to provide a comprehensive perspective on nutritional status; however, they rely on a limited set of components and may not fully encapsulate overall nutritional health. Research on colorectal cancer reveals mixed results regarding the prognostic value of these indices [10, 31]. For instance, lower PNI has been linked to higher rates of postoperative complications and reduced survival [32], although some studies indicate that the connection between PNI and complications is not always clear [33, 34]. These limitations underscore the necessity for a more thorough assessment of nutritional status.

TRG and ypN status are well-established prognostic markers in LARC [35–37]. TRG assesses tumor response to preoperative therapy and correlates with pathological complete response. Improved TRG is generally associated with decreased local recurrence and enhanced survival outcomes. Various TRG systems exist, all indicating that patients exhibiting significant regression experience better outcomes compared to those with minimal regression [38]. ypN status is among the strongest predictors post-neoadjuvant therapy, with ypN-positive patients consistently showing earlier recurrence than their node-negative counterparts [39]. ypN positivity also indicates a heightened risk of distant metastasis and necessitates consideration for intensified adjuvant therapy. Studies that integrate TRG and ypN demonstrate superior risk stratification compared to either measure alone [40]. In clinical practice, the combination of these metrics leads to more accurate prognostic models, aiding in decisions regarding adjuvant treatment and surveillance intensity.

Patients undergoing NCRT often face increased metabolic demands and treatment-related toxicities. Maintaining an adequate nutritional reserve is crucial for tolerating treatment, minimizing unplanned interruptions, and supporting postoperative recovery [41, 42]. Early identification of patients with compromised nutritional status can enable clinicians to provide timely support and mitigate the risk of adverse events. Although the present study was not designed to assess the effects of nutritional interventions, identifying patients with low CNI may help generate hypotheses for targeted nutritional support, intensified monitoring, or tailored supportive care strategies in future prospective studies. In this context, CNI serves as a practical tool that captures multiple dimensions of nutritional and physiological condition. By integrating CNI with TRG and ypN status, we have developed a prognostic nomogram with potential clinical applicability and straightforward interpretability. This nomogram facilitates individualized survival estimation and may assist clinicians in patient counseling, planning supportive care, and customizing follow-up strategies. Such an approach can enhance risk stratification in the context of multimodal treatment for LARC. Notably, CNI is derived from routinely available pre-treatment clinical parameters, enabling it to function as an early risk indicator prior to neoadjuvant therapy. Conversely, the proposed nomogram incorporates postoperative pathological variables, such as tumor regression grade and nodal status, and is primarily intended for prognostic stratification after surgery rather than for pre-treatment decision-making.

This study has several limitations. First, its retrospective, single-center design and the inclusion of only patients with complete data introduce potential selection bias and limit generalizability. External validation was not conducted, and the sample size was modest. Second, nutritional status was assessed at a single predetermined time point, failing to capture dynamic changes throughout treatment. Third, postoperative complications were not systematically analyzed, as the primary focus was on long-term survival outcomes. Additionally, several relevant factors were not included in the analysis, such as postoperative adjuvant chemotherapy, inflammatory markers, molecular characteristics, and surgical approach. Notably, emerging evidence suggests that robotic total mesorectal excision may be associated with improved oncological outcomes in selected patients with locally advanced rectal cancer, particularly in males with mid- to low-rectal tumors [43–45]. Therefore, the exclusion of surgical technique may represent a potential confounding factor and should be considered when interpreting the results. Collectively, these limitations indicate that while CNI appears to be an independent prognostic indicator in patients with LARC treated with neoadjuvant chemoradiotherapy, further studies incorporating external validation cohorts and direct comparative analyses with other nutritional indices are warranted to elucidate its relative performance and clinical utility.

## Conclusion

In conclusion, pretreatment CNI represents a practical and informative nutritional composite associated with prognosis in patients with locally advanced rectal cancer treated with neoadjuvant chemoradiotherapy. Risk stratification using CNI may aid clinicians in identifying patients with varying prognostic profiles and support more individualized therapeutic planning.

## Supplemental data

**Table S1:** Baseline clinicopathological characteristics categorized by CNI group

**Characteristics**	**High-CNI (*n* ═ 192)**	**Low-CNI (*n* ═ 144)**	***P* value**
Age (years)	58.51 (9.84)	53.60 (11.45)	<0.001
Gender (female/male)	64 (33.3)/128 (66.7)	48 (33.3)/96 (66.7)	1.000
BMI	26.08 (2.47)	21.39 (1.86)	<0.001
Smoking history (yes/no)	118 (61.5)/74 (38.5)	87 (60.4)/57 (39.6)	0.846
Drinking history (yes/no)	125 (65.1)/67 (34.9)	95 (66.0)/49 (34.0)	0.868
Hypertension (yes/no)	123 (64.1)/69 (35.9)	117 (81.2)/27 (18.8)	<0.001
Diabetes (yes/no)	150 (78.1)/42 (21.9)	136 (94.4)/8 (5.6)	<0.001
cTNM (II/III)	26 (13.6)/165 (86.4)	26 (18.1)/118 (81.9)	0.266
ypTNM (I/II/III)	35 (18.2)/93 (48.4)/64 (33.3)	27 (18.8)/70 (48.6)/47 (32.6)	0.988
Vessel invasion (yes/no)	178 (92.7)/14 (7.3)	126 (87.5)/18 (12.5)	0.108
Perineural invasion (yes/no)	167 (87.0)/25 (13.0)	117 (81.2)/27 (18.8)	0.151

Data are presented as means (standard deviations) for continuous variables and as counts (percentages) for categorical variables. Student’s *t*-test was used to calculate *P* values for continuous variables, whereas the chi-square test or Fisher’s exact test was applied to categorical variables, as appropriate. It is important to note that due to missing data, the totals for some variables may not sum to the overall cohort size. Abbreviations: CNI: Comprehensive nutritional index; BMI: Body mass index; cTNM: Clinical tumor–node–metastasis stage; ypTNM: Postoperative pathological tumor–node–metastasis stage.

## Data Availability

De-identified data and code used for model training are available upon reasonable request to the corresponding author, in accordance with institutional and national data protection regulations.
